# Tick-Borne Encephalitis in an 8.5-Month-Old Boy Suspected of Febrile Seizures

**DOI:** 10.3390/microorganisms9071425

**Published:** 2021-07-01

**Authors:** Lenka Krbková, Iva Čapovová, Lukáš Homola, Jana Lindušková, Jiří Salát, Daniel Růžek

**Affiliations:** 1 Department of Children’s Infectious Diseases, Faculty of Medicine and University Hospital, Masaryk University, Jihlavská 100, CZ-62500 Brno, Czech Republic; capovova.iva@fnbrno.cz (I.Č.); homola.lukas@fnbrno.cz (L.H.); 2 Department of Microbiology and Immunology, University Hospital, Jihlavská 100, CZ-62500 Brno, Czech Republic; linduskova.jana@fnbrno.cz; 3 Laboratory of Emerging Viral Infections, Veterinary Research Institute, Hudcova 70, CZ-62100 Brno, Czech Republic; salat@vri.cz (J.S.); ruzekd@paru.cas.cz (D.R.); 4 Institute of Parasitology, Biology Center, Czech Academy of Sciences, 37005 Ceske Budejovice, Czech Republic

**Keywords:** tick-borne encephalitis, CNS infection, child, paediatric infections

## Abstract

Tick-borne encephalitis (TBE) is a serious viral neuroinfection affecting humans in large areas of Europe and Asia. TBE can occur at any age, but only a few reports of TBE in infants younger than 1 year have been published. Here, we report a case of severe TBE in an 8.5-month-old boy presenting with seizures at the beginning of the neurological phase.

## 1. Introduction

Tick-borne encephalitis (TBE) is a viral infection of the central nervous system transmitted by infected ticks. The disease is endemic across many areas of Europe, including the Czech Republic, and extends to large parts of Asia [1]. The Czech Republic has one of the highest incidences of TBE in Europe [2]. No antiviral drug is available against TBE virus (TBEV), but prophylactic vaccination is an effective measure. TBE vaccination is currently recommended for endemic areas, including children ≥1 year old (WHO). The Czech Republic is one of the European countries where the vaccination rate against TBE remains low [1].

TBE is a cause of neurological involvement in children, predominantly manifested as an abortive form or meningitis. Infants are rarely affected with meningoencephalitis. Only a few cases of TBE in children up to the age of one year in Europe have been reported [3,4,5,6,7,8]. Here, we present a case of TBE in an 8.5-month-old boy presenting with seizures at the beginning of the neurological phase.

## 2. Case Report

The 8.5-month-old boy spent a lot of time in the garden, where ticks were present. The habitat is highly endemic for TBE. The parents and two older siblings are completely vaccinated against TBE. No genetic disorder or severe illness was mentioned in the family history. The pregnancy was normal and physiological delivery was at term. The boy had been breastfed to the fourth month of life; thereafter, meat, vegetables, and fruits were added. He was vaccinated against diphtheria, tetanus, pertussis, poliomyelitis, hepatitis B, and *Haemophilus influenzae* type b according to the mandatory schedule. In addition, vaccines against rotaviral infection and 13-valent pneumococcal conjugated vaccine were administered. Somatic and mental development did not show any abnormalities. No regular medication was taken. A tick bite in the left inguinal region with no local reaction was reported 16 days before admission. One week after the tick bite, 4-day transitory subfebrile episodes were referred by the mother and diagnosed as exanthema subitum by the pediatrician despite no skin rash. After a three-day afebrile period, a maximum rectal temperature of 40 °C was recorded. Symmetric seizures pronounced in the lower extremities and unconsciousness of approximately 1-min duration were described by the parents. Respiration and cardiac function were maintained. After the attack, the boy was hypotonic, and he was transported to a local hospital.

The boy was admitted with the suspected diagnosis of febrile seizures. After a repeated short episode of generalized seizures without febrile status, he was transferred to the university hospital. Physical examination did not show any pathological findings except for a fever of 38.1 °C. He remained febrile for five days and irritable. Extensive laboratory tests were performed. Complete blood count examination showed leucocytosis of 27.52 × 10^9^/L (normal count 6–17.5 × 10^9^/L), of which 53.5% were neutrophils, 37.8% lymphocytes, 6.2% monocytes, and 1.3% eosinophils. Hemoglobin was decreased to 98 g/L (normal level 105–135 g/L), and the platelet count was elevated to 917 × 10^9^/L (normal count 150–450 × 10^9^/L). The sedimentation rate was 65/91 in 1/2 h. C-reactive protein was 27.6 mg/L (normal value 0–5 mg/L). Serum biochemistry was normal except for slightly elevated alanine aminotransferase (0.69 µkat/L; normal value 0.2–0.63 µkat/L).

At his first neurological examination, no pathology was found except for a bulging fontanelle and neck stiffness. Lumbar puncture and electroencephalography were recommended. Analysis of the cerebrospinal fluid (CSF) revealed pleocytosis (440/µL leukocytes with 70% predominance of lymphocytes) and an increased protein concentration of 0.93 g/L (normal value 0.15–0.45 g/L). CSF glucose and lactate were normal. Microbiological analysis of herpetic viruses (HSV1/2, VZV, and HHV6) was performed by polymerase chain reaction (PCR). The results were negative in both the CSF and serum. Antibodies against *Borrelia burgdorferi* were tested in serum and CSF by ELISA. Potential blood-brain barrier disruption was determined. In line with recommended standards, the IgM and IgG antibody index was assessed. Both indices were negative for the intrathecal synthesis of antiborrelial antibodies. CSF culture was negative. ELISA found antibodies against TBEV in the CSF (IgM 11.9 and IgG 3.8) and serum (IgM 5.8 and IgG 2.2) (the index of positivity is borderline from 0.9 to 1.1, and positive result is >1.1). As a gold standard for specific serological diagnosis of TBE, a virus neutralization test (VNT) was performed [9]. The Czech prototype TBEV strain Hypr was used in the assay. The titer of specific anti-TBEV neutralization antibodies in sera was 1:80. Thus, a definitive diagnosis of TBE was made.

The electroencephalograph revealed no epileptiform activity and no lateralization. The organized activity was detected predominantly with theta waves and delta waves sporadically corresponding with the patient’s age. Treatment was started with intravenous ceftriaxone (100 mg/kg per day) and dexamethasone (0.5 mg/kg per day) for five days. After Lyme neuroborreliosis was excluded and TBE proven, ceftriaxone treatment was stopped. No further convulsions were noted, and the gentle tremor in the upper extremities disappeared after three days.

He was discharged home with no detectable sequelae. The patient was re-examined at our department three times during the subsequent 10-month period. Neurological follow-up showed no abnormalities, and no regression was seen in his mental and motor development. Laboratory findings were normal. Antibodies against TBEV were positive in serum (IgG 5.2 and IgM 1.3) at his last follow-up control. The neutralization titer increased at his first control one month later to 1:320 and a half year later to 1:640.

## 3. Discussion

In children, TBE infection is described as having a mild clinical course. TBE under the age of three years is rare, and the mean age of the meningoencephalitic form of TBE is 14 years [10]. The reason why childhood TBE is usually less severe than TBE in adults is still not fully understood. A literature search revealed only five case reports of infants with TBE during the first year of life [4,5,6,7,8], including one in the Czech Republic [5].

The disease may result in permanent central nervous system sequelae, including cognitive complaints characteristic of postencephalitic syndrome, in children with slow progression of mental and motor development [11].

The history of a tick bite, biphasic course of the infection, and clinical findings together with CSF analysis in our patient hinted at aseptic meningitis. The initial suspicion of febrile seizures usually caused by HHV6 infection in this age group was excluded by the negative HHV6 PCR result in CSF and negative serum antibodies. Repeated seizures in an afebrile episode of the illness suggested that the definitive diagnosis is TBE. Antibodies against TBEV in the CSF, as well as in the serum, confirmed the TBEV infection. Therefore, TBE diagnosis should be considered during routine workup, even in infants with neurological signs.

Both European vaccines (FSME-IMMUN, Pfizer, and Encepur, GSK Vaccines), more specifically their pediatric formulations (FSME-IMMUN Junior or Encepur Children), are approved for use with children aged from 1 to 15 years (using FSME-IMMUN Junior) and from 1 to 11 years (Encepur Children) [1]. The case reported here provides further information for recent discussions regarding whether the TBEV vaccination should be extended to children under one year of age and living in highly endemic areas for TBE. In addition, the parents should be aware that their children can be infected with TBEV, and the infection may also happen in city parks or their own gardens.

## Data Availability

The data that supports the findings of this study are available from the corresponding author upon reasonable request.
